# miR-429 suppresses cell proliferation, migration and invasion in nasopharyngeal carcinoma by downregulation of TLN1

**DOI:** 10.1186/s12935-019-0831-0

**Published:** 2019-04-30

**Authors:** Zhihui Wang, Zhiquan Zhu, Zhong Lin, Youli Luo, Zibin Liang, Caibin Zhang, Jianxu Chen, Peijian Peng

**Affiliations:** 1 Department of Thoracic Oncology, The Cancer Center of The Fifth Affiliated Hospital of Sun Yat-sen University, 52 Meihua East Road, Zhuhai, Guangdong China; 2 Department of Neurology, The Fifth Affiliated Hospital of Sun Yat-sen University, 52 Meihua East Road, Zhuhai, Guangdong China; 3 Department of Hepatobiliary Surgery, The Fifth Affiliated Hospital of Sun Yat-sen University, 52 Meihua East Road, Zhuhai, Guangdong China

**Keywords:** Nasopharyngeal carcinoma, miR-429, TLN1

## Abstract

**Background:**

miR-429 and TLN1 have been shown to affect the biological behaviours of many carcinomas. However, their effects in nasopharyngeal carcinoma (NPC) are not yet clear. Here, we investigated their regulatory relationships and effects on NPC cells.

**Methods:**

TargetScan was used to predict the regulatory relationships of miR-429 and TLN1 in NPC cells. Then, Western blotting and quantitative real-time PCR (qPCR) were performed to examine TLN1 levels, and qPCR was used to determine miR-429 levels in NPC cell lines with different metastatic characteristics (5-8F, CNE-2, CNE-1, 6-10B and NP69), to investigate whether TLN1 and miR-429 are correlated with the metastatic characteristics of these cells. Next, we upregulated or downregulated miR-429 in 5-8F and 6-10B cells, which have different tumourigenicity and transferability, and examined TLN1 expression by western blotting and qPCR after transfection. QPCR was also performed to confirm successful transfection of miR-429 mimic into 5-8F and 6-10B cells. Dual luciferase reporter gene assay was performed to investigate whether miR-429 regulates TLN1 by binding to its 3′UTR. After transfection, Cell Counting Kit-8 (CCK8) and IncuCyte were used to examine the proliferation of these cells, and wound-healing assay, Transwell migration assay, and invasion assays were performed to investigate the changes in migration and invasion after transfection.

**Results:**

Western blotting and qPCR analyses showed that the protein level of TLN1 was negatively correlated with miR-429 in NPC cell lines (*P *< 0.05), while the mRNA level showed no relation with miR429 expression (*P *> 0.05). In addition, cells with high transferability showed high TLN1 expression at the protein level, while miR429 expression showed the opposite trend (*P *< 0.05), but there were no differences at the mRNA level between the different cell lines. Overexpression of miR429 in 5-8F and 6-10B cells was accompanied by downregulation of TLN1 at the protein level (*P *< 0.05), while there were no significant differences at the mRNA level (*P *> 0.05). In addition, transferability, proliferation, and invasion were downregulated by miR429 overexpression (*P *< 0.05). However, dual-luciferase reporter gene assay indicated that TLN1 was not a direct target of miR-429.

**Conclusion:**

This study showed that miR-429 functions as a tumour suppressor in NPC by downregulation of TLN1, although the relationship is not direct.

## Background

Nasopharyngeal carcinoma (NPC) is a malignancy derived from the nasopharyngeal epithelium and consists of three pathological subtypes according to the WHO criteria: keratinising squamous cell carcinoma, non-keratinising carcinoma, and non-keratinising carcinoma (differentiated subtype) [1]. In addition, NPC shows clear regional differences in its occurrence; according to the statistics of GLOBALCAN 2018, there were an estimated 129,079 new cases of NPC worldwide in 2018, with approximately 90% of new cases occurring in economically less developed countries, with the highest incidence rates in South-Eastern Asia, where Malaysia, Indonesia, Singapore, South-Eastern China (including Guangdong and Hong Kong) and other parts of Southern Asia (the Philippines, India and Thailand) have very high incidence rates [2, 3]. Epstein–Barr virus (EBV) infection is believed to be one important risk factor for NPC. In addition, genetic susceptibility and consumption of preserved foods, such as salted fish, etc., are believed to be closely related to the occurrence of NPC [3, 4]. Radiotherapy is the primary treatment for patients with early-stage NPC, and concurrent chemoradiotherapy is the standard treatment for patients with locoregionally advanced NPC (LA-NPC). However, up to one-third of LA-NPC patients die from distant metastasis [5, 6]. Therefore, it is necessary to identify novel markers for treating NPC patients with distant metastasis.

MicroRNAs (miRNAs) are endogenous, small non-coding RNAs about 22 nucleotides (nt) in length, which can regulate gene expression by binding to the 3′-untranslated region (3′UTR) of target mRNAs for degradation or translational repression. Some miRNAs have been shown to be related to many physical and pathological processes, including cell death, cell proliferation, tumourigenesis, tumour metastasis, etc. [7–10]. In NPC, several miRNAs have been shown to regulate cell proliferation, invasion, migration, apoptosis, radioresistance, etc. Some act as tumour suppressors, while others are promoters. For example, miR-204, miR-124, miR-152, miR-185, miR-342, miR-3423p, miR-379, miR-425, miR-429, miR-203a-3p, miR-34a and miR-148a are tumour suppressors [11–22], while miR-142-3p, miR-222, miR-92a, miR-93, miR-346 and miR-663 are tumour promoters [23–28]. miR-429 is a member of the miR-200 family, which has been shown to act as a tumour suppressor in many carcinomas, including bladder cancer, gastric cancer, soft tissue sarcoma, cervical cancer, renal cell carcinoma, glioblastoma, osteosarcoma, and hepatocellular carcinoma [29–38], while this miRNA plays an oncogenic role in colorectal cancer and non-small cell lung cancer (NSCLC) [39, 40]. miR**-**429 was shown to be expressed at low levels in metastatic lesions of NPC patients and to inhibit proliferation, invasion and migration of some NPC cell lines by targeting zinc finger E-box-binding homeobox 1 (ZEB1) and CRK-like (CRKL) protein expression [19, 41].

TLN1 is a cytoskeletal protein with a weight of about 270 kDa, which is vital for the activation of integrin, and which promotes linkage between integrins and actin [42–45]. To date, high TLN1 expression level has been shown to boost migration and invasion of various carcinomas, such as NPC, prostate cancer and glioblastoma, while it showed the opposite effect in hepatocellular carcinoma [46–50].

Here, we investigated the regulatory relationship between miR-429 and TLN1 in NPC cell lines. Further, we examined the influence of miR-429 on the biological behaviours of NPC cell lines.

## Materials and methods

### NPC cell lines and cell culture

All NPC cell lines used in this study (5-8F, CNE-2, CNE-1, 6-10B and NP69) were established and donated by Sun Yat-sen University Cancer Center. The NPC cell lines (5-8F, CNE-2, CNE-1 and 6-10B) were cultured in RPMI-1640 medium (Gibco, Grand Island, NY, USA) supplemented with 10% foetal bovine serum (Gibco). The immortalised nasopharyngeal epithelial cell line NP69 was cultured in Defined Keratinocyte Serum-Free Medium (Catalogue no. 10744019; Gibco). All cell lines were incubated at 37 °C with 5% CO_2_.

### miRNA target gene prediction

TargetScan [51] was used to predict potential targets of miR-429. After entering the website, select “human” in the species box, then input “TLN1” in the human gene symbol box, afterwards, input “hsa-miR-429” in the microRNA name box and click “submit”, we can get their relevant predictions.

### Western-blot

Total proteins of each cell line were extracted with PMSF-containing RIPA lysate buffer (Solarbio, Beijing, China), and the concentrations of which were detected with BCA kit (Beyotime, Shanghai, China), afterwards, total proteins were mixed with loading 5× buffer (Solarbio, Beijing, China) and heated to 100 °C for 5 min and stored at − 20 °C until used. Total proteins were separated in 8% SDS-PAGE gels and transferred onto polyvinylidene fluoride membranes (Milipore, USA). The membranes were blocked in TBST with 5% skimmed-milk, and incubated with rabbit monoclonal anti-Talin-1 (C45F1) antibody (1:1000; CST, USA) at 4 °C over night, then incubated with anti-rabbit IgG secondary antibody (1:1000; Beyotime, Shanghai, China). β-actin was used as loading control. The bands were detected in ChemiDoc™ XRS + System (Bio-Rad, USA) using enhanced chemiluminescence. All assays were performed in triplicate.

### Real-time fluorescent quantitative PCR assay

Total RNA was extracted with Trizol (Invitrogen, Carlsbad, CA, USA), trichloromethane, isopropanol and 75% ethanol (diluted with RNase-free water), and the concentration was determined using a UV–Vis spectrophotometer (NanoDrop 2000; Thermo Fisher Scientific, Waltham, MA, USA). The A260/280 value was confirmed to range from 1.8 to 2.0. The cDNAs of miR-429 and U6 were reverse transcribed in a T100™ Thermal Cycler (Bio-Rad, Hercules, CA, USA) with an RT reagent Kit (RR037A; Takara, Otsu, Japan) according to the manufacturer’s instructions. Real-time fluorescent quantitative PCR assay was performed using a CFX96™ Real-Time PCR Detection System (Bio-Rad, Hercules, CA, USA) with TB Green™ Premix Ex Taq™ (RR420A; Takara, Otsu, Japan). The PCR conditions were as follows: 95 °C for 30 s, followed by 39 cycles of 95 °C for 5 s, 60 °C for 30 s then go to Melt Curve. U6 was used as a loading control. The miR-429 primers were designed and synthesised by SangonBiotech (Shanghai, China); the RT primer sequence was 5′-gtc gta tcc agt gca ggg tcc gag gta ttc gca ctg gat acg aca cgg-3′, and the reverse primer sequence was 5′-agt gca ggg tcc gag gta tt-3′. The RT and reverse primers of U6 were designed and synthesised by Ribobio Inc. (Guangzhou, China). All assays were performed in triplicate.

### Transfection with miRNA mimics

The miR-429 mimic, anti-miR-429 and their negative controls were purchased from Ribobio Inc. The high transferability NPC cell line 5-8F and low transferability NPC cell line 6-10B were seeded into 6-well plates at a density of 2 × 10^5^ cells/well. After 24 h, transfections were performed using Lipofectamine 3000 (Invitrogen, Carlsbad, CA, USA) according to the manufacturer’s protocol. The final concentrations of miR-429 mimic and its negative control were 50 nM, while those of anti-miR-429 and its negative control were 30 nM. The transfected cells were incubated at 37 °C with 5% CO_2_, and the subsequent experiments were performed 48 h after transfection. All assays were performed in triplicate.

### Dual luciferase reporter assays

The 3′-UTR wild-type and mutated sequences of TLN1 were synthesised and inserted into the primiR-RB-Report™ vector (Ribobio Inc. Guangzhou, China). HEK 293T cells were co-transfected with 50 nM miR-429 mimic and 500 ng/mL plasmid. Forty-eight hours later, the Dual-Glo^®^Luciferase Assay System (Promega, Madison, WI, USA) was used to examine the effects of miR-429 on TLN1 according to the manufacturer’s protocol. All assays were performed in triplicate.

### Cell proliferation assay

Twenty-four hours after transfection, 5-8F and 6-10B were harvested with trypsin and seeded into 96-well plates at a density of 1000 cells per well. Their absorbance at 450 nm was examined after reseeding and incubation for 0, 24, 48, 72 or 96 h using CCK8 (Dojindo, Kumamoto, Japan) according to the manufacturer’s protocol. IncuCyte (Essen BioScience, Ann Arbor, MI, USA) was used to detect cell proliferation. The assays were performed in triplicate.

### Cell migration and invasion assay

Twenty-four hours after transfection, 5-8F and 6-10B cells were harvested with trypsin and seeded into Transwell chambers (Catalog no. 3422; Corning Inc., Corning, NY, USA) at a density of 1 × 10^4^ cells per well. The upper chambers contained 100 uL of 0.1% FBS-containing RPMI-1640 medium, while the lower chambers contained 600 uL of 10% FBS-containing RPMI-1640 medium. Incubation was performed for 24 h (invasion assay, 50 uL Matrigel was added to the Transwell chamber at a concentration of 200 ug/mL before seeding of cells, and incubated for 48 h after seeding). The upper chambers were removed from the lower chambers and the untransfected cells and Matrigel were wiped off the upper chambers using cotton swabs. The invaded cells were fixed with 4% paraformaldehyde for 30 min and dyed with 1% crystal violet for 30 min at room temperature. The numbers of migrated cells were counted in five random fields of view under a microscope at 50× magnification (Leica, Wetzlar, Germany). The experiment was repeated independently three times.

### Wound-healing assay

Twenty-four hours after transfection, a wound was made by scratching, using a ruler and a 200 uL pipette tip. The medium was removed and washed twice gently with PBS. Then 1% FBS-containing RPMI-1640 medium was added and incubation was performed at 37 °C with CO_2_. Photographs were taken under a microscope at 50× magnification at 0, 24, 48 and 72 h after wounding. The assay was repeated independently three times.

### Statistical analysis

The results are presented as mean ± SEM. The data were analysed using Student’s *t* test or one-way ANOVA depending on the characteristics of the data. IBM SPSS Statistics version 20 (IBM, Armonk, NY, USA) was used for statistical analyses. In all analyses, *P *< 0.05 was taken to indicate statistical significance.

## Results

### TLN1 is a potential target of miR-429

**Fig. 1:**
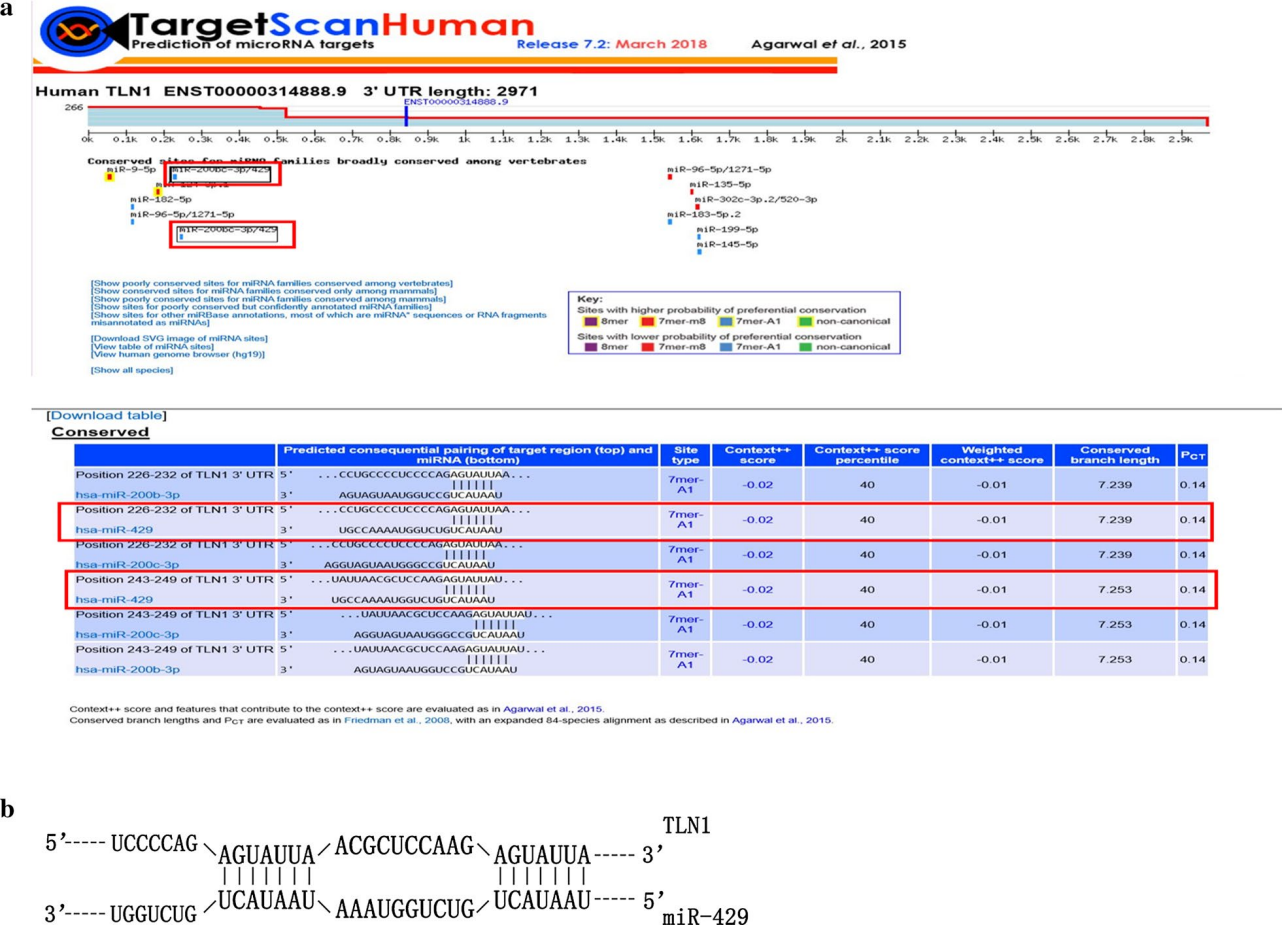
Prediction of TargetScan. **a** The predicted regulatory relationships and scores between miR-429 and TLN1 at TargetScan; **b** the binding sites of TLN1 and miR-429

TargetScan predicted that TLN1 was a potential target of miR-429, with two potential binding sites and a context ++ score percentile of 40 (Fig. 1).

### TLN1 protein is highly expressed in highly metastatic NPC cell line, while no difference was observed in its mRNA level

**Fig. 2:**
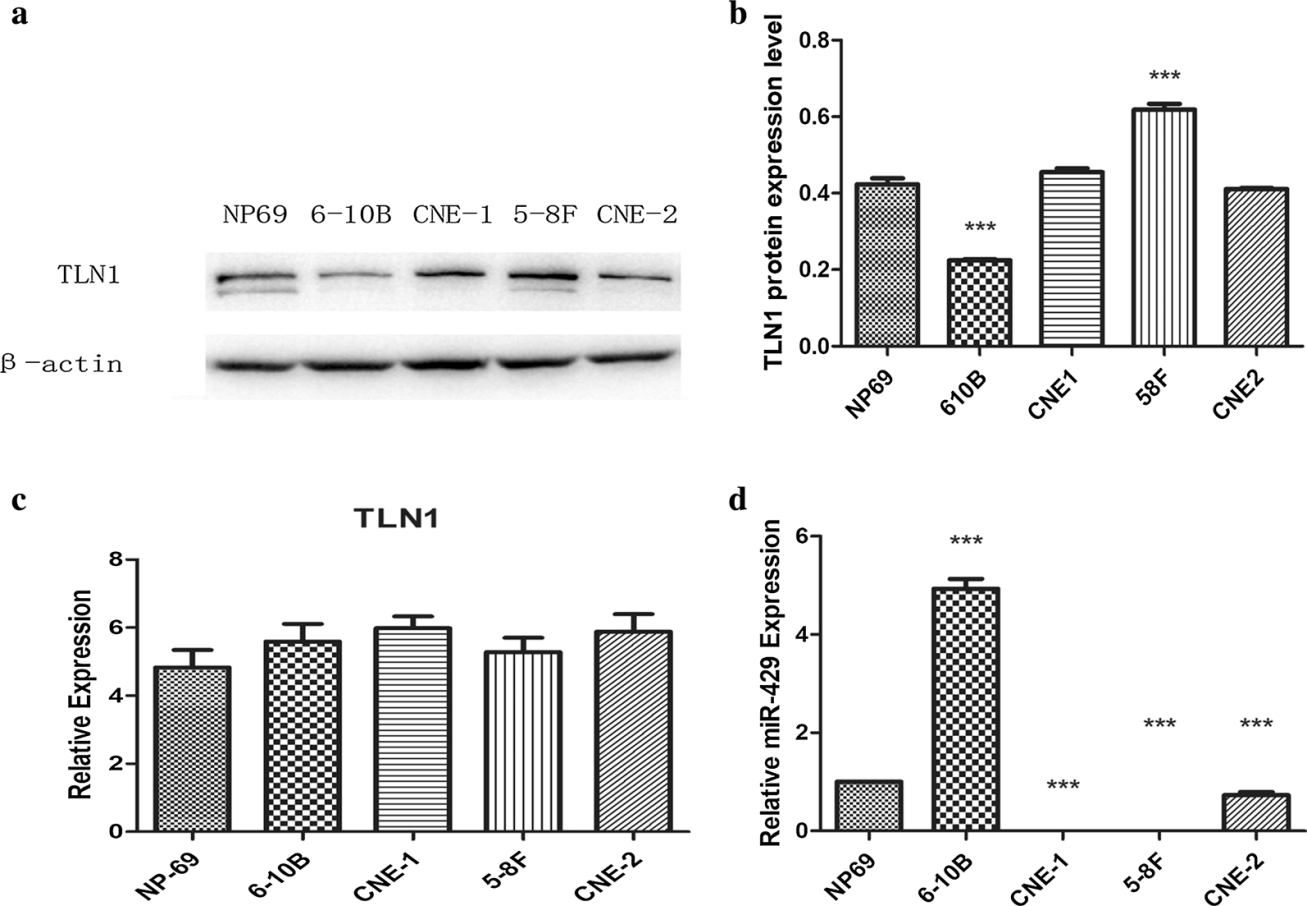
Detections of TLN1 and miR-429 expression profiles in human NPC cell lines. **a–c** Relative expression profiles of TLN1 in 4 NPC cell lines (CNE-2, 5-8F, CNE-1, 6-10B) and immortalized nasopharyngeal epithelial cell line (NP69); **d** relative expression profiles of miR-429 in 4 NPC cell lines (CNE-2, 5-8F, CNE-1, 6-10B) and immortalized nasopharyngeal epithelial cell line (NP69). All data are presented as mean ± SD, *P < 0.05, **P < 0.01, ***P < 0.001

Western blotting and qPCR were used to measure the protein and mRNA levels in NPC cell lines (5-8F, CNE-2, CNE-1, 6-10B and NP69). The results indicated that TLN1 was highly expressed at the protein level in 5-8F (Fig. 2a, b; *P *< 0.05), which is highly metastatic, and showed low levels of expression in 6-10B (Fig. 2a, b; *P *< 0.05), which has low metastatic potential. There were no statistically significant differences in expression at the mRNA level between the five cell lines (Fig. 2c; *P *> 0.05).

### miR-429 is highly expressed in NPC cell line with low metastatic potential

We used qPCR to measure the levels of miR-429 in NPC cell lines (5-8F, CNE-2, CNE-1, 6-10B and NP69). The results indicated that miR-429 was highly expressed in NP69 and 6-10B, which have low transferability, while the levels of expression in 5-8F, CNE-2 and CNE-1, which have high transferability, were low (Fig. 2d; *P *< 0.05).

### miR-429 was successfully transfected into NPC cells

**Fig. 3:**
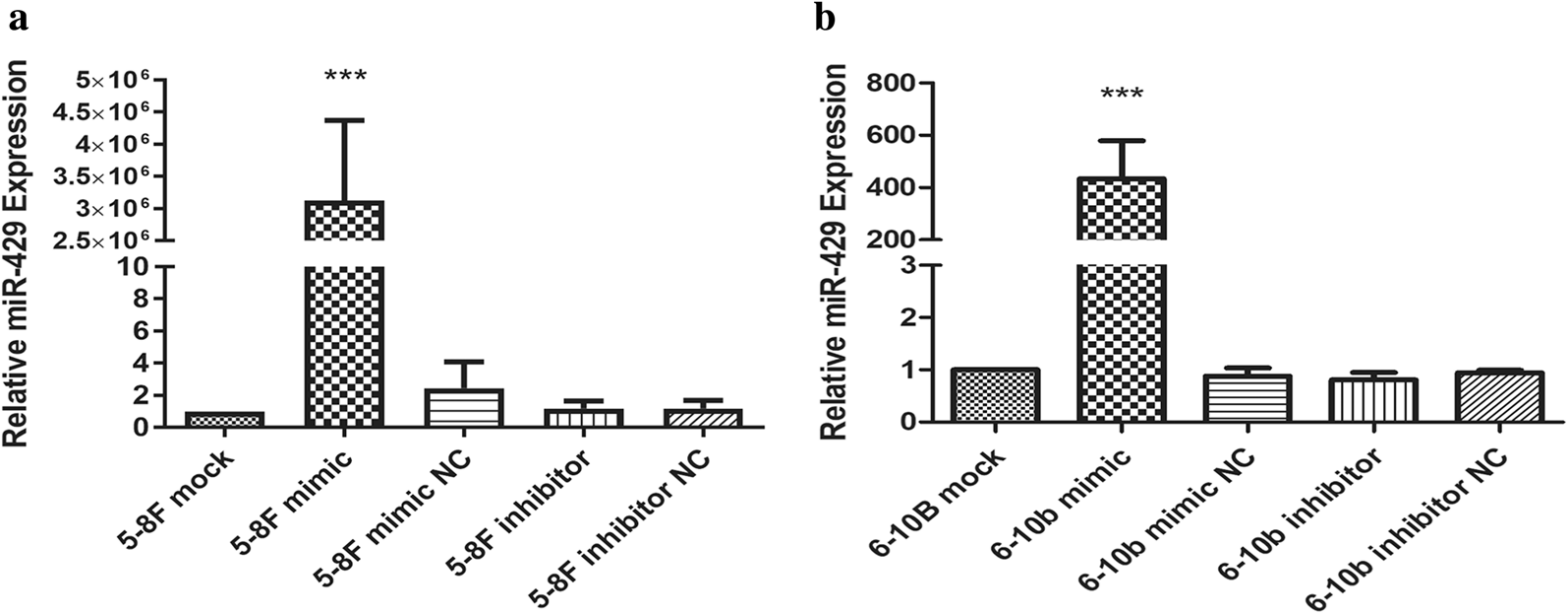
Transfection efficiencies of miR-429 mimic in NPC cell lines. **a** The expression levels of miR-429 in 5-8F after being transfected with miR-429 mimic, miR-429 mimic negative control, miR-429 inhibitor and miR-429 inhibitor negative control for 48 h; **b** the expression levels of miR-429 in 6-10B after being transfected with miR-429 mimic, miR-429 mimic negative control, miR-429 inhibitor and miR-429 inhibitor negative control, all data are presented as mean ± SD, *P < 0.05, **P < 0.01, ***P < 0.001

To investigate the regulatory effects of miR-429, we transfected miR-429 mimic and miR-429 inhibitor into 5-8F and 6-10B to upregulate and downregulate miR-429. Their negative controls were used as controls. QPCR was used to detect the transfection efficiency. After transfection, miR-429 was markedly upregulated in mimic groups (Fig. 3a, b; *P *< 0.05), while no differences were observed in the others (Fig. 3a, b; *P *> 0.05).

### TLN1 protein was downregulated by miR-429

**Fig. 4:**
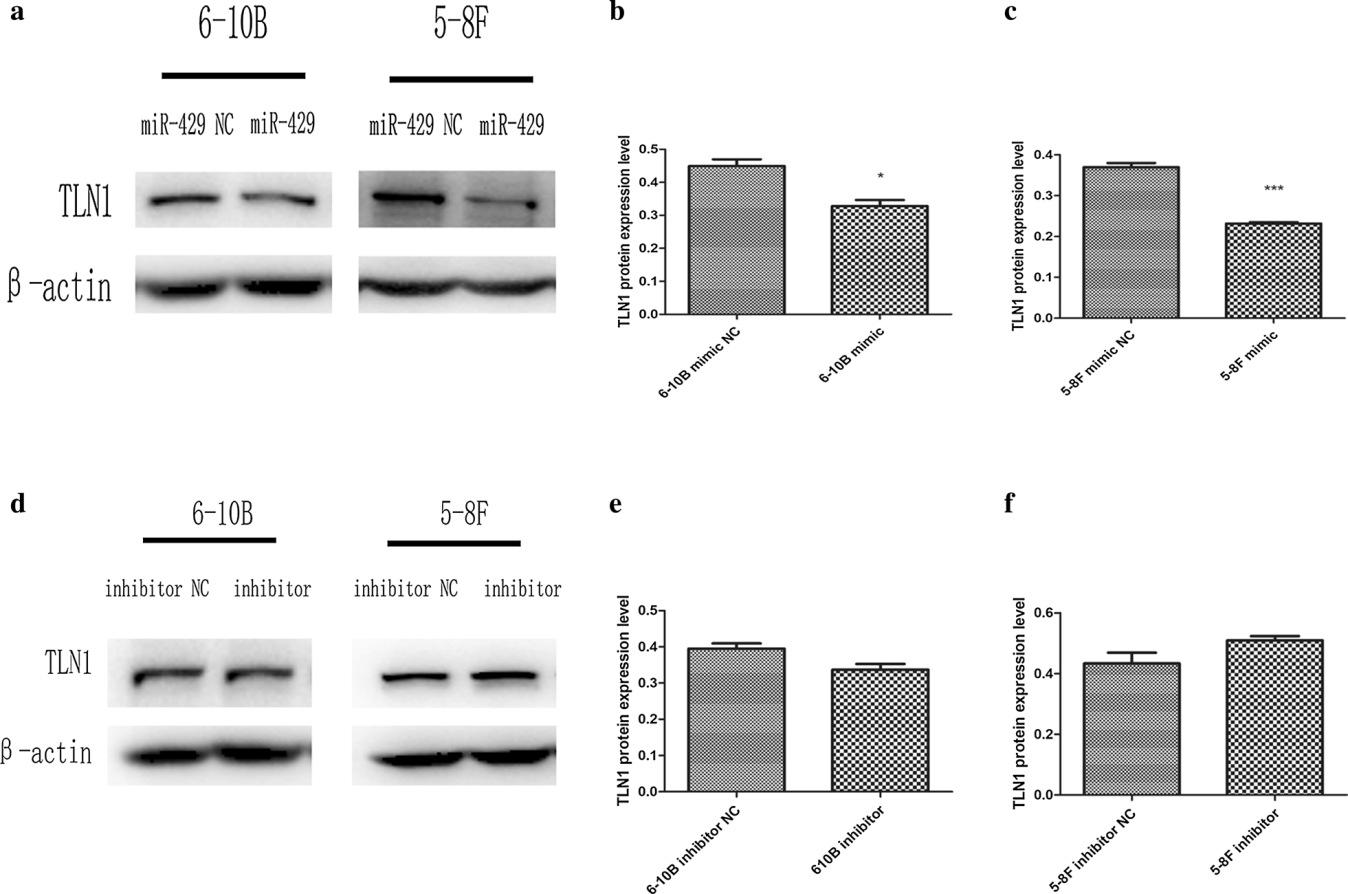
Effects of miR-429 on protein levels of TLN1. **a**–**f** Western-blot and gray value were used to measure the effects of miR-429 on protein levels of TLN1 in 5-8F and 6-10B, all data are presented as mean ± SD, *P < 0.05, **P < 0.01, ***P < 0.001

**Fig. 5:**
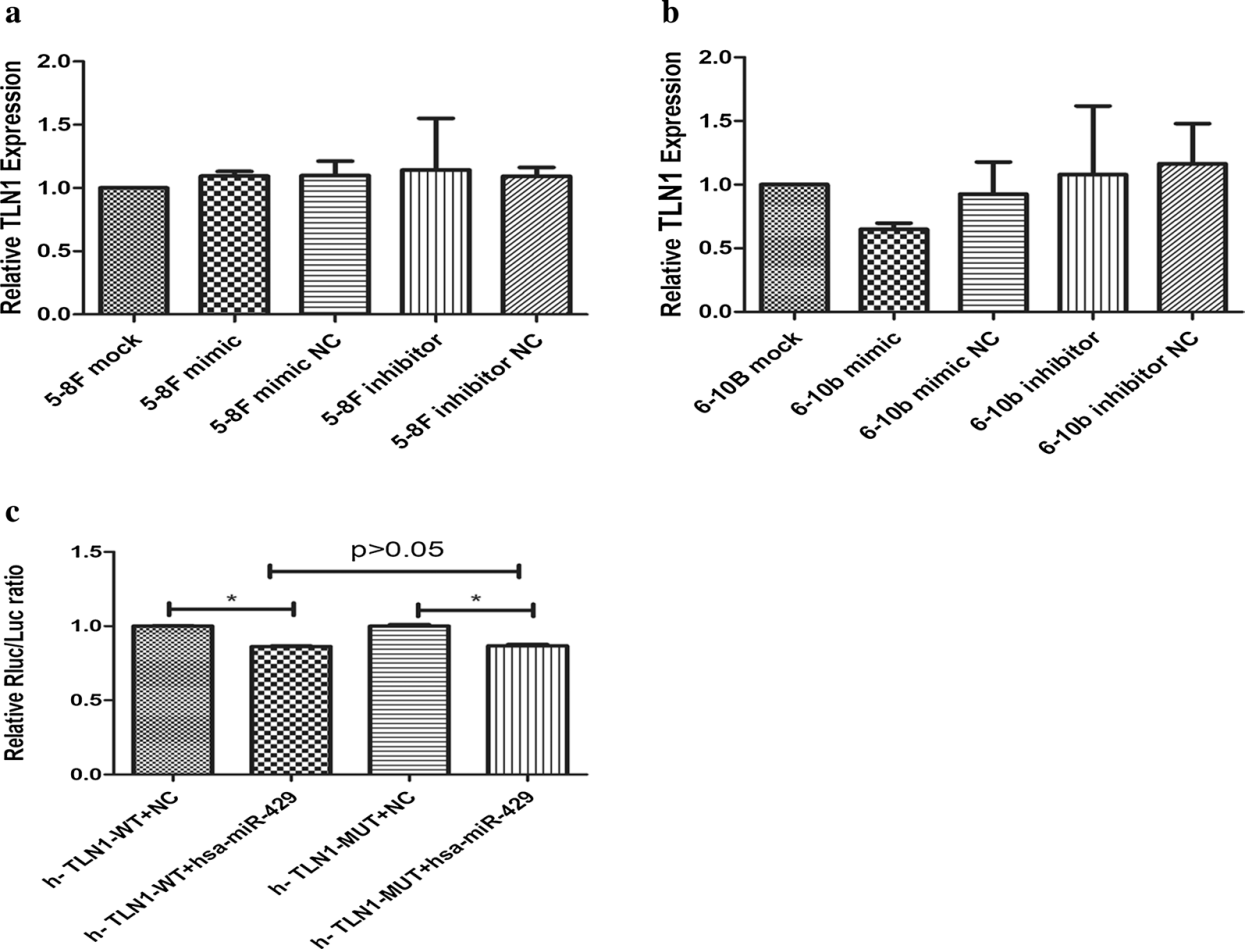
Effects of miR-429 on mRNA levels of TLN1 and target verification. **a**, **b** Q-PCR was used to measure the effects of miR-429 on mRNA levels of TLN1 in 5-8F and 6-10B; **c** dual-luciferase reporter assay was used to verify whether TLN1 is a target of miR-429. All data are presented as mean ± SD, *P < 0.05, **P < 0.01, ***P < 0.001

To investigate the regulatory relationships between TLN1 and miR-429, we transfected miR-429 mimic and miR-429 inhibitor into 5-8F and 6-10B to upregulate and downregulate miR-429. After transfection, qPCR and western blotting analyses were performed to measure the expression of TLN1 at the mRNA and protein levels. Western blotting analysis showed that TLN1 was downregulated by miR-429 mimic in both 5-8F and 6-10B (Fig. 4a–c; *P *< 0.05), and it was upregulated in 5-8F and downregulated in 6-10B by miR-429 inhibitor, but there were no significant differences comparing to the control groups in both cell lines (Fig. 4d–f; *P *> 0.05). In addition, qPCR showed that the levels of TLN1 mRNA were not significantly different among these groups after transfection in both cell lines (Fig. 5a, b; *P *> 0.05).

### TLN1 is not a direct target of miR-429

The results of dual-luciferase reporter assays showed that, in both h-TLN1-WT + hsa-miR-429 and h-TLN1-MUT + hsa-miR-429 groups, the relative Rluc/Luc ratios were downregulated relative to the controls (Fig. 5c). These observations indicated that TLN1 is not a direct target of miR-429.

### miR-429 suppresses 5-8F cell proliferation in vitro

**Fig. 6:**
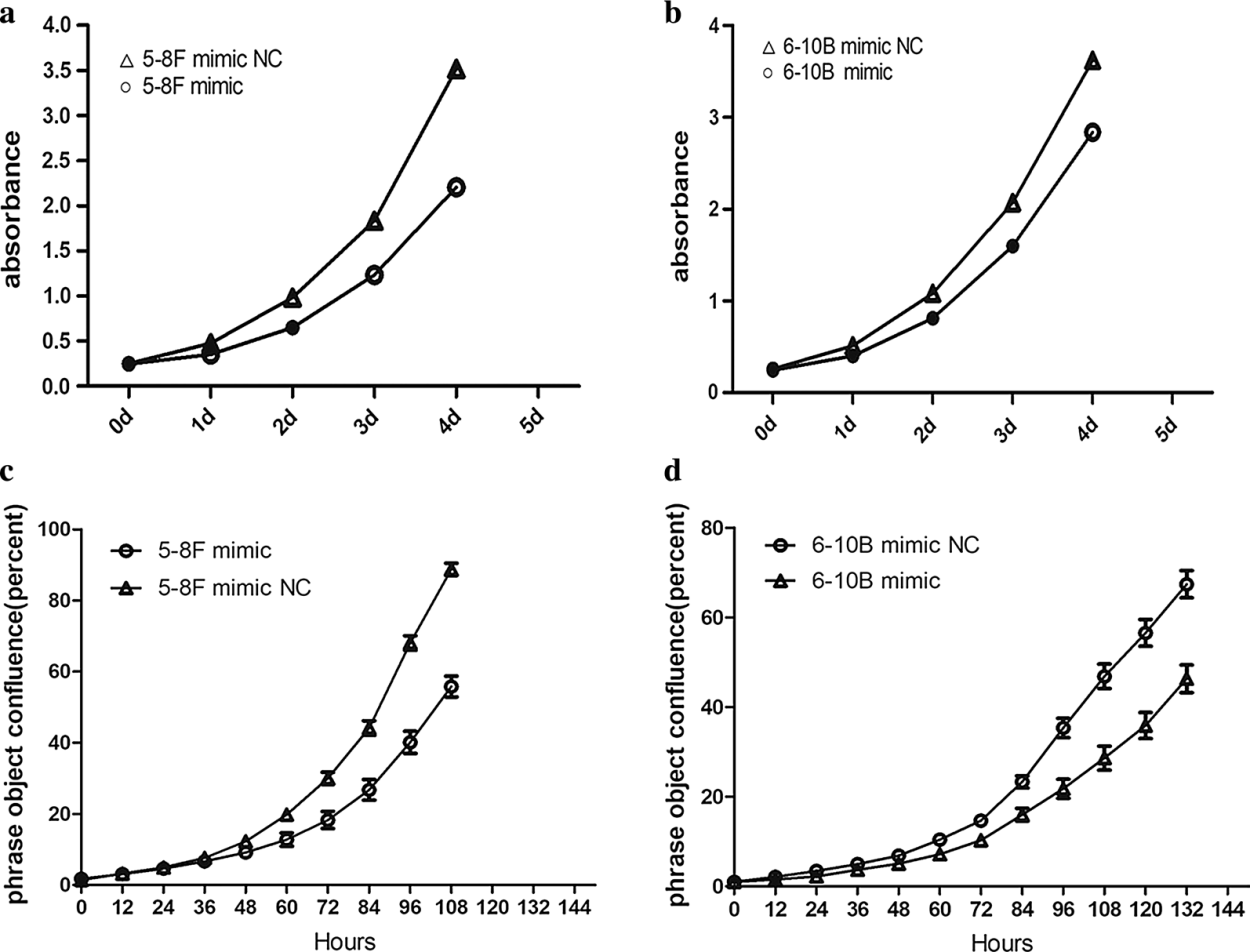
Effects of miR-429 overexpression on proliferation of NPC cell lines. **a** The absorbance of CCK8 after being transfected with miR-429 mimic and miR-429 mimic negative control in 5-8F; **b** the absorbance of CCK8 after being transfected with miR-429 mimic and miR-429 mimic negative control in 6-10B; **c** phrase object confluence of 5-8F after being transfected with miR-429 mimic and miR-429 mimic negative control; **d** phrase object confluence of 6-10B after being transfected with miR-429 mimic and miR-429 mimic negative control; all data are presented as mean ± SD, *P < 0.05, **P < 0.01, ***P < 0.001

To investigate whether miR-429 influences the proliferation of NPC cell lines, we used CCK8 and a live cell dynamic imaging and analysis system to measure the proliferation of 5-8F and 6-10B after transfection with miR-429 mimic and its negative control. The miR-429 mimic inhibited the proliferation of both cell lines compared to negative controls, but the difference was statistically significant for 5-8F, while it was not significant for 6-10B (Fig. 6a–d).

### miR-429 inhibits the migration and invasion of NPC cells

**Fig. 7:**
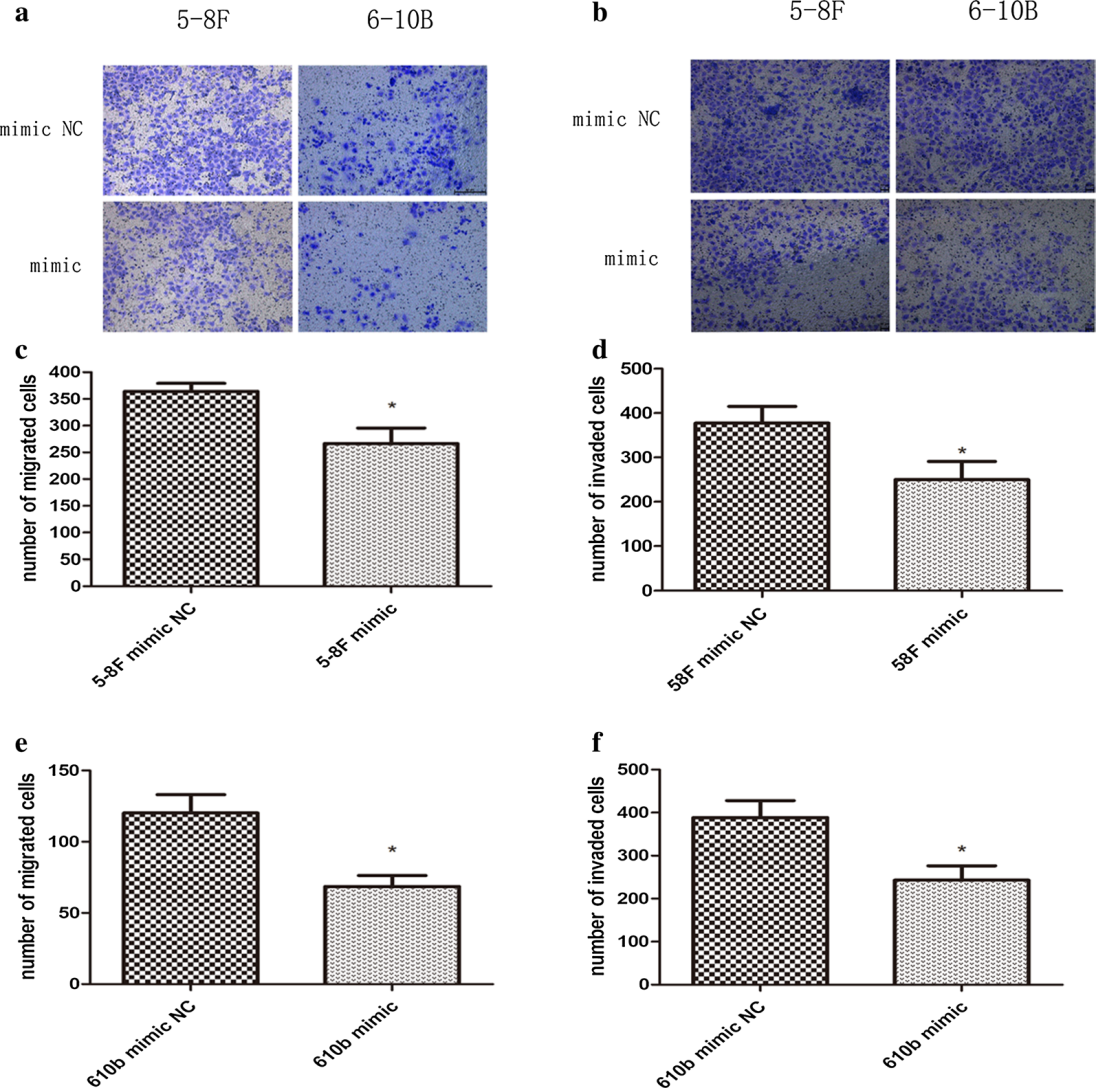
Effects of miR-429 overexpression on migration and invasion of NPC cell lines. **a**, **c**, **e** Results of transwell migration assay of 5-8F and 6-10B. **b**, **d**, **f** Results transwell invasion assay of 5-8F and 6-10B. All data are presented as mean ± SD, *P < 0.05, **P < 0.01, ***P < 0.001

**Fig. 8:**
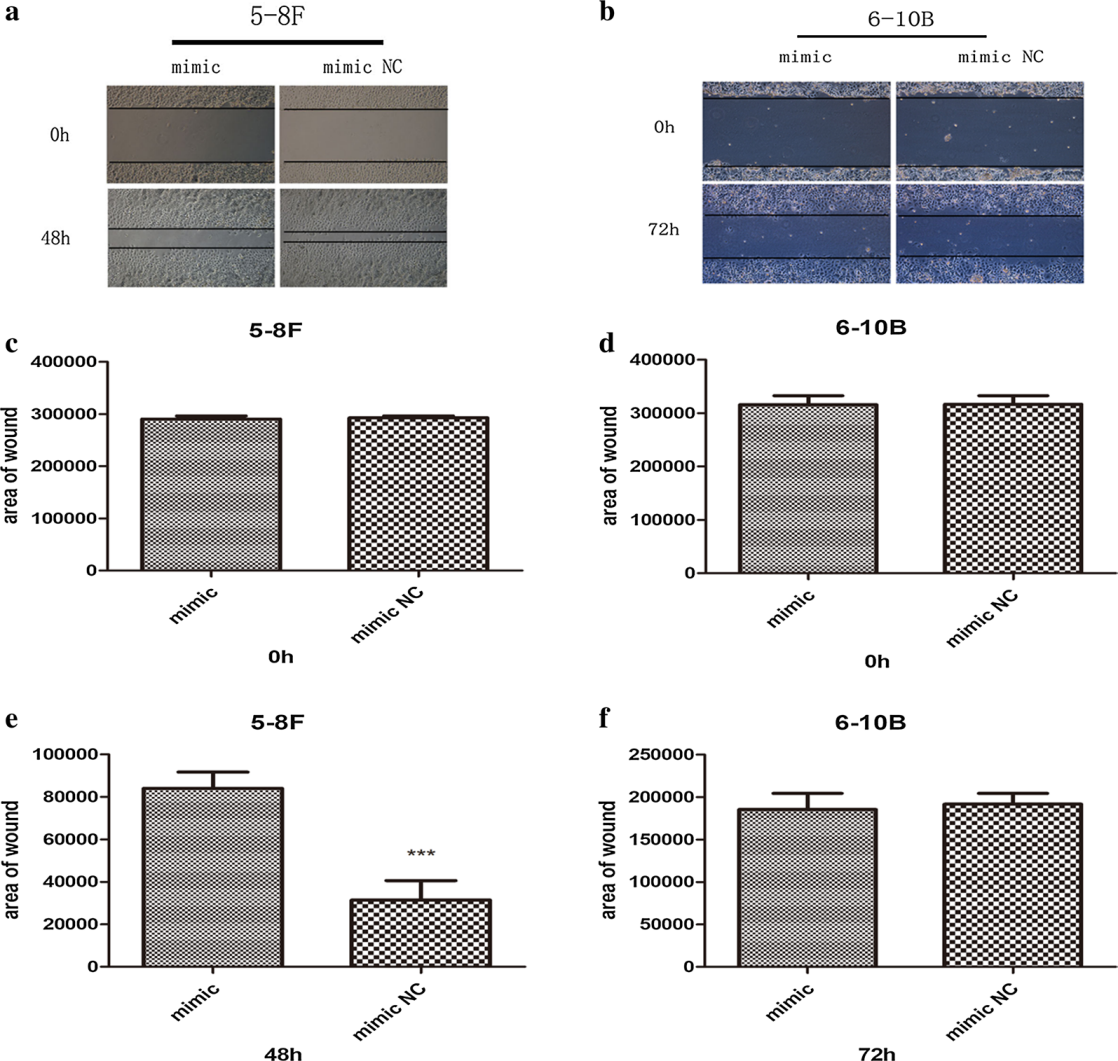
Effects of miR-429 overexpression on wound-healing assay of NPC cell lines. **a**–**f** Results of wound-healing assay of 5-8F and 6-10B. All data are presented as mean ± SD, *P < 0.05, **P < 0.01, ***P < 0.001

Both 5-8F and 6-10B showed significant suppression of migration and invasion by miR-429 mimic in Transwell assays (Fig. 7a–f). In addition, 5-8F showed significant suppression of wound healing by miR-429 mimic (Fig. 8a, c, e). However, both mimic and control groups showed similar migration and invasion activities for 6-10B (Fig. 8b, d, f).

## Discussion

As described above, many miRNAs have been shown to act as tumour promoters or suppressors in various cancers, and they may represent novel targets in cancer treatment. In NPC, some miRNAs have been reported to be tumour suppressors or promoters, which may be significant in patients with advanced disease. An in silico analysis revealed the miRNA expression profiles of NPC at different stages, which suggested that these miRNAs may be novel targets to treat patients with advanced NPC [41]. MiR-429 was shown to be expressed at low levels in metastatic lesions, and was demonstrated to be a tumour suppressor gene in NPC [19, 41]. To confirm its targets and mechanism of action in NPC, we used TargetScan (http://www.targetscan.org) to predict the potential targets of miR-429. The results suggested that TLN1 may be a target of miR-429 (Fig. 1). TLN1 is a cytoskeletal protein, which was shown to be a tumour promoter in NPC [46]. Therefore, we postulated that miR-429 may act as tumour suppressor by targeting TLN1 in NPC.

We investigated the correlations between miR-429 and TLN1, and their differences among different NPC cell lines. We found that miR-429 was negatively correlated with metastatic potential among NPC cell lines (Fig. 2d). At the mRNA level, there were no significant significances in TLN1 expression among these cell lines relative to their own β-actins. However, at the protein level, TLN1 expression was significantly higher in 5-8F and lower in 6-10B (Fig. 2a–c). These results were consistent with those of previous studies [19, 46]. In summary, we showed that miR-429 and TLN1 expression are negatively correlated in NPC cell lines. To confirm their regulatory relationships, we transfected miR-429 mimic and its inhibitor into 5-8F and 6-10B cells to upregulate or downregulate miR-429. qPCR was used to measure the transfection efficiency. Our results showed that miR-429 was markedly upregulated in the mimic groups, while there were no significant effects in the other groups (Fig. 3a, b), as for inhibitor groups, the mechanism of which is competitive inhibition, so it will not affect the level of miR-429. Subsequently, we found that TLN1 expression was not significantly changed at the mRNA level, in either cell line (Fig. 5a, b). At the protein level, however, TLN1 was significantly downregulated in mimic groups, but there were no significant differences in inhibitor groups (Fig. 4a–f). These results suggested that TLN1 may be a target of miR-429, and its expression may be downregulated at the protein level by miR-429. However, dual-luciferase reporter assay showed that TLN1 is not a direct target of miR-429 (Fig. 5c).

Although the results of dual-luciferase reporter assay indicated that TLN1 is not a direct target of miR-429, the protein level of TLN1 was downregulated by miR-429 mimics, suggesting that miR-429 may regulate TLN1 through other mechanisms. Further studies are required to investigate this issue.

After transfection, we performed a series of assays to examine the effects of miR-429 on the biological behaviours of NPC cells. After transfection, qPCR showed that the levels of miR-429 were not significantly downregulated by inhibitor, maybe owing to the mechanism of which is competitive inhibition. Therefore, we subsequently only investigated the biological effects of the mimic groups relative to the negative controls. Our functional results suggested that miR-429 can suppress proliferation of NPC cells (Fig. 6a–d), although the effect was not marked, and it can inhibit the migration and invasion capabilities of NPC cells (Figs. 7a–f, 8a, c, e), consistent with previous studies [19]. Taken together with the results reported previously [46], our observations suggested that miR-429 functions as a tumour suppressor via downregulation of TLN1 in NPC.

## Conclusion

In summary, the results of the present study indicated that miR-429 inhibits proliferation, migration and invasion of NPC cells by downregulating TLN1, although the effect was not mediated by a direct regulatory relationship. Increasing numbers of miRNAs have been shown to regulate the biological behaviours of many tumours, including NPC. Therefore, miRNAs may be useful as novel therapeutic targets, and our observations provide suggestions for future treatment of NPC.

## Abbreviations

NPC: nasopharyngeal carcinoma
EBV: Epstein–Barr virus
LA-NPC: locoregionally advanced NPC
3′UTR: 3′-untranslated region
NSCLC: non-small cell lung cancer
qPCR: real-time quantitative reverse transcription-polymerase chain reaction
